# Immunotherapy for NAFLD and NAFLD-related hepatocellular carcinoma

**DOI:** 10.3389/fendo.2023.1150360

**Published:** 2023-03-20

**Authors:** Seogsong Jeong, Woo-Young Shin, Yun Hwan Oh

**Affiliations:** ^1^ Department of Biomedical Informatics, CHA University School of Medicine, CHA University, Seongnam, Republic of Korea; ^2^ Institute for Biomedical Informatics, School of Medicine, CHA University, Seongnam, Republic of Korea; ^3^ Department of Family medicine, Chung-Ang University Gwangmyeong Hospital, Chung-Ang University College of Medicine, Gwangmyeong, Republic of Korea

**Keywords:** non-alcoholic fatty liver disease, immune system, immunity, liver cancer, chronic liver disease

## Abstract

The progression of non-alcoholic fatty liver disease (NAFLD), the most common liver disease, leads to non-alcoholic steatohepatitis and hepatocellular carcinoma. Despite the increasing incidence and prevalence of NAFLD, its therapeutic and preventive strategies to lower the disease burden is limited. In recent years, immunotherapy, including anti-programmed cell death 1/programmed cell death 1 ligand 1 treatment, has emerged as a potential approach to reach satisfactory modulation for the progression of NAFLD and treatment of NAFLD-related hepatocellular carcinoma. However, the effectiveness of immunotherapy against NAFLD and NAFLD-related hepatocellular carcinoma is in the early phase and it is yet not advanced. In addition, conflicting results are being reported regarding the prognosis of patients with NAFLD-related hepatocellular carcinoma and high expression of programmed cell death 1/programmed cell death 1 ligand 1. Herein, this review will discuss and elucidate the attempts and underlying mechanisms of immunotherapy against NAFLD and NAFLD-related hepatocellular carcinoma.

## Introduction

Non-alcoholic fatty liver disease (NAFLD) has reached a global prevalence of 30% and the increasing trend is ongoing (1). It involves a spectrum of diseases, including hepatic steatosis and non-alcoholic steatohepatitis (NASH) (2). NAFLD may lead to life-threatening hepatic diseases, such as liver cirrhosis and hepatocellular carcinoma (HCC), and extrahepatic diseases, such as cardiovascular disease and dementia (3–6). Along with advances in retrospective operational definitions, such as fatty liver index and Korea National Health and Nutrition Examination Survey-NAFLD score, and non-invasive diagnostic approaches for NAFLD, prevention, management, and treatment of NAFLD are being actively investigated (7–9).

In recent years, NAFLD-related HCC (NAFLD-HCC) has emerged as a major factor contributing to the disease’s burden (10). In addition, NAFLD-HCC in non-cirrhotic patients is increasingly identified (11). The major risk factors of NAFLD-HCC include liver cirrhosis, old age, male sex, patatin-like phospholipase domain-containing 3 variants, diabetes, and obesity (12). Epigenetic factors, including transcriptional factors and post-transcriptional modifications, individual-level characteristics, and environmental factors have been reported to be associated with the development and progression of NAFLD-HCC (13).

Up to date, the identified potential therapeutic options for NAFLD include herbal medicine, a low-calorie diet, physical activity, polyphenol, bile acid, anti-inflammatory agents, hormones, and pre and probiotics, as confirmed in a clinical trial (14–19). However, recent findings are suggesting that immunotherapy is promising against NAFLD, NASH, or NAFLD-HCC. In this study, we review the recent findings regarding the effects of immunotherapy, especially programmed cell death 1 (PD-1) and programmed cell death 1 ligand 1 (PD-L1) treatment against NAFLD, NASH, or NAFLD-HCC.

## Current treatment options for NAFLD and NASH

Considering the close relationship between diabetes and NAFLD, a number of antidiabetic medications were testified as a therapeutic approach for NASH in an experimental setting (10). Glucagon-like peptide 1 (GLP1) is a hormone that stimulates the release of insulin, indirectly suppresses the secretion of glucagon, and lowers food intake (20). Currently, a number of G protein-coupled GLP1 receptor (GLP1R) agonists, including dulaglutide, exenatide, liraglutide, and semaglutide, are approved as therapeutics for diabetes (20). GLP1R agonist ameliorated liver damage and hepatic steatosis in diet-induced NASH mice (21). In addition, treatments with exenatide, liraglutide, and semaglutide have indicated their efficacy in the reduction of hepatic lipid contents and the level of liver enzymes (22–24). Since the expression level of GLP1R is not high, its effect may be due to systemic modification in metabolism rather than direct amelioration of the liver (25). Dipeptidyl peptidase 4 (DPP4), which inactivates GLP1, inhibitors have also been reported to reduce liver fibrosis and the development of liver tumors in the mouse NASH model (26). However, sitagliptin was found not effective in improving NAFLD activity score or fibrosis score, suggesting that DPP4 inhibitors may not be promising in the treatment of NAFLD and NASH (27). In addition, a murine liver cancer model study has identified that liraglutide has the potential to promote the anti-tumor effects of PD-1 inhibition through the reduction of neutrophil extracellular traps in liver cancer (27).

Thiazolidinedione, including rosiglitazone and pioglitazone, has been suggested to modify the sensitivity of peripheral insulin through stimulation of the adipokine release, enhancing the inhibitory effects of insulin on lipolysis and leading to a reduced plasma level of free fatty acids, as well as lowered accretion of hepatic lipids (28). A previous study also reported that thiazolidinedione reduces the activation of hepatic stellate cells and liver fibrosis in rats (29). Rosiglitazone was previously reported to be effective in enhancing insulin sensitivity and reducing hepatic steatosis, but there were cases of weight gain and edema in the lower extremity (30, 31). In addition, the efficacy of sodium-glucose cotransporter 2 (SGLT2) has been testified in NAFLD, and found that dapagliflozin and empagliflozin treatment modifies liver fat and liver enzyme levels (32, 33). Apart from antidiabetic treatment, nuclear receptor modulators, *de novo* lipogenesis inhibitors, and fibroblast growth factors were also suggested as potential therapeutic approaches (10).

Recently, obeticholic acid, an agonist of farnesoid X receptor (FXR) has emerged a potential therapeutic approach for adult NASH patients with at least 4 NAFLD activity score and fibrosis stages of F2 to F3 or F1 with comorbidity (34). The improvement in fibrosis was significantly detected in the obeticholic acid 25 mg group (n=71; 23%) compared to the placebo group (n=37; 12%) in a phase 3 trial. In addition, the efficacy and safety of another FXR agonist, tropifexor, has more recently been tested for NASH in a phase 2 trial (35). Dose-related pruritus was detected, and decreases in hepatic fat fraction and alanine aminotransferase were sustained until 48^th^ week. Therefore, FXR agonists may be the closest treatment option against NAFLD and NASH.

## Immunotherapy for NAFLD and NASH

The immune system is expected to play an important role in the development, modulation, and progression of NAFLD. To date, potential targets to prevent the progression of NAFLD have been selected, including molecules that express on the immune system cell surface, and the PD-1/PD-L1 complex is receiving attention (36). Within the liver, PD-1, which is a membrane protein that is exposed by all T cells, responds against lymphocyte activation through PD-L1, of which its upregulation is supported by interferon-γ (37).

Cenicriviroc first showed its targeting ability for proinflammatory monocytes through the dual chemokine 2 and chemokine 5 receptor antagonists in a murine model, then it was found to improve liver fibrosis in patients with biopsy-proven NAFLD in a phase 2b clinical trial (38). However, the subsequent phase 3 clinical trial could not reach the primary endpoint, which is an improvement of liver fibrosis in a condition of not worsening NASH. Another phase 2b trial is evaluating the efficacy of cenicriviroc with Tropifexor, which is a farnesoid X receptor agonist that is involved in lipid metabolism and bile acid synthesis, for NASH (NCT03517540) (39). However, the study results are yet unpublished in the literature.

Peroxisome proliferator-activated receptor (PPAR) agonists-mediated and exerted anti-inflammatory effects *via* targeting macrophages were also tested in trials (40). The activation of PPARs regulates lipid metabolism and inflammation *via* the modulation of macrophage in tissues, including the liver. However, elafibranor, which is a PPAR agonist, revealed no significant benefits for liver histology in the phase 3 trial (40). Another phase 2 clinical trial evaluated the efficacy of a murine monoclonal antibody targeting T cell receptor-associated CD3 (41). In addition, a human anti-CD3 antibody (foralumab) was developed and tested in a phase 2 clinical trial (NCT03291249) for patients with NASH and diabetes. However, this trial was withdrawn due to a request from the Ministry of Health.

The upregulation of PD-1, which is a membrane protein that is exposed by all T cells, is modulated by interferon-γ, and the inhibition of PD-1/PD-L1 T cells is modulated by interfering with the T-cell receptor/CD28 signal, leading to reduced production of pro-inflammatory cytokines (36). PD-1 was upregulated after the interleukin-15 treatment-medicated downregulation of forkhead box protein O1 in mice CD8+ T cells, and the level of interleukin-15 was found to be associated with high C-X-C chemokine receptor type 6+PD-1-high CD8+ T cells and low expression of forkhead box protein O1 (36, 42). In addition, PD-1/PD-L1 may express in dendritic cells, which are exposed to intestinal pathogen-associated molecular patterns to decelerate immune responses, to prevent inflammation (43). However, despite a number of attempts and efforts in controlling the progression and pathogenesis of NAFLD by modulating the immune system, no conclusive evidence has been provided.

## Immunotherapy for NAFLD-HCC

**Figure 1:**
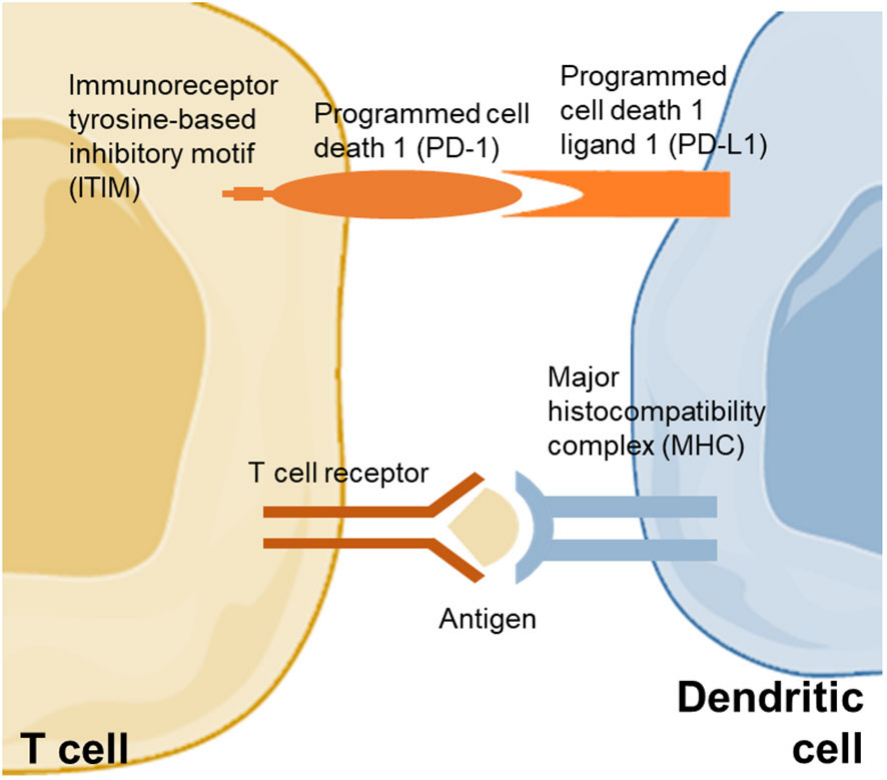
PD-1 and PD-L1 between dendritic cell and T cell. PD-1 pathway activation is modulated by the binding of Src homology domain-containing protein tyrosine phosphatase 1 and 2 to immunoreceptor tyrosine-based inhibitory motif that repress the proliferation of T cell. The pathway modulates inhibitory signals for the activation of T cell.

PD-1 interferes with protective immune responses and contributes to the expansion of malignant cells (44). PD-L1, which can prevent the proliferation of tumor-specific T cells *via* suppressive signals, leading to impaired anti-tumor immunity, is commonly expressed in malignant cells (**Figure 1**) (45). Polymorphisms in PD-1 have been found associated with a higher risk of cancers (44). The rs10204525 and rs7421861 variants boosted the expression of PD-1 and were found associated with a higher risk of esophageal cancer in the Asian population (46). A multi-European cohort of 391 NAFLD-HCC patients found that the PD-1 rs7421861 variant is associated with NAFLD-HCC in the United Kingdom population only, suggesting that there may be a difference in PD-1-related risk of NAFLD-HCC according to ethnicity (47). In an Egyptian cohort of 134 NASH and NASH-related HCC patients, the PD-L1 rs2282055 variant was associated with the risk of cancer (48). In another study of 167 patients with HCC, the level of PD-L1 was increased within the liver and was positively correlated with interferon-γ (49). Notably, patients with a higher expression of PD-L1 and CD8+ had better survival compared to those with a lower expression of PD-L1 and CD8+, indicating that PD-L1 and CD8+ cytotoxic T cells may promote the eradication of HCC. Another study also confirmed that a lower expression of PD-L1 and CD8+ tumor-infiltrating lymphocytes predicted poor HCC-specific survival in patients after liver resection (50). However, there were a few studies that demonstrated a higher expression of PD-L1 as an unfavorable factor for the prognosis of patients with HCC, including the recurrence of HCC after resection (51, 52).

To date, a number of pharmaceutical agents that produces monoclonal PD-1 receptor and prevents the escape of tumor cells *via* blockage of the PD-1 system are developed, including pembrolizumab and nivolumab (**Table 1**) (53–59). In the phase 2 clinical trial of pembrolizumab, the effectiveness and tolerance were satisfactory, suggesting that pembrolizumab may be a treatment option for patients with advanced HCC after sorafenib treatment (53). In addition, Feun et al. (54) also showed similar results and further supported that the toxicity was tolerable and reversible. In the phase 3 clinical trial of pembrolizumab, statistically significant improvement in overall survival and progression-free survival was identified but the statistical significance was insufficient for the pre-specified criteria (55). However, the phase 3 clinical trial of pembrolizumab in Asian patients demonstrated that pembrolizumab significantly improved overall survival (hazard ratio [HR], 0.79; 95% confidence interval [CI], 0.63-0.99; P=0.018), progression-free survival (HR, 0.74; 95% CI, 0.60-0.92; P=0.003), and objective response rate (P<0.001). As for nivolumab versus sorafenib, nivolumab had a higher median overall survival (HR, 0.85; 95% CI, 0.72-1.02; P=0.075) but the predefined significance level of P=0.0419 was not achieved (57).

**Table 1:** Phase 2 to 3 clinical trials of immunotherapy against HCC.

Author	Clinical trial No.	Phase	No. of patients	Intervention	Result
Zhu et al. (53)	NCT02702414	2	104	Pembrolizumab	Complete response: 1 (1%) Partial response: 17 (16%)
Feun et al. (54)	NCT02658019	2	28	Pembrolizumab	Complete response: 1 (4%) Partial response: 8 (29%)
Finn et al. (55)	NCT02702401	3	413	Pembrolizumab vs. placebo	Median OS: 13.9 months vs. 10.6 months Median PFS: 3.0 months vs. 2.8 months
Qin et al. (56)	NCT03062358	3	453	Pembrolizumab vs. placebo	Median OS: 14.6 months vs. 13.0 months Median PFS: 2.6 months vs. 2.3 months
Yau et al. (57)	NCT02576509	3	743	Nivolumab vs. sorafenib	Median OS: 15.2 months vs. 13.4 months
Finn et al. (58)	NCT03434379	3	501	Atezolizumab plus bevacizumab vs. sorafenib	Median PFS: 6.8 months vs. 4.3
Abou-Alfa (59).	NCT03298451	3	1,171	Tremelimumab plus durvalumab vs. durvalumab vs. sorafenib	Median OS: 16.4 months vs. 16.6 months vs. 13.8 months Complete response: 12 (3.1%) vs. 6 (1.5%) vs. 0 Partial response: 67 (17.0%) vs. 60 (15.4%) vs. 20 (5.1%)

Acronyms: OS, overall survival; PFS, progression-free survival.

In addition, atezolizumab, which selectively targets PD-L1 and reserves T-cell suppression, plus bevacizumab, which targets vascular endothelial growth factor and inhibits angiogenesis, was compared with sorafenib in the treatment of unresectable HCC (58). The risk of death was significantly lower in the atezolizumab plus bevacizumab group compared to sorafenib group (HR, 0.58; 95% CI, 0.42-0.79; P<0.001). Furthermore, Abou-Alfa et al. (59) testified tremelimumab (cytotoxic T lymphocyte–associated antigen 4 inhibitor) plus durvalumab (anti–PD-L1) or durvalumab monotherapy versus sorafenib in patients with unresectable HCC, which indicated significant improvement in overall survival after tremelimumab plus durvalumab compared to sorafenib and non-inferiority of durvalumab monotherapy was identified compared to sorafenib.

## Conclusion

The immune system and related pathways play crucial roles in the progression and pathogenesis of NAFLD. Despite recent accumulating attention in immunotherapy against NAFLD, the literature remains insufficient to make a conclusive estimation on whether immunotherapy, especially targeting PD-1/PD-L1, may prevent NASH or HCC in patients with HCC. In addition, promising results are being reported and new pharmaceutical immunotherapy is being developed and tested in clinical trials. However, most of these clinical trials testified the efficacy of immunotherapy in patients with unresectable or advanced HCC, thus the results may not represent therapeutic effectiveness for all NAFLD-HCC cases. In addition, since non-viral HCC, especially NASH-HCC, may be less responsive to immunotherapy potentially due to the activation of NASH-related aberrant T cell that causes tissue damage and subsequent impaired immune surveillance, immunotherapy resistance may be one of the most critical challenges (60). Understanding the underlying mechanisms of the resistance in immunotherapy against NAFLD, NASH, or NAFLD-HCC may provide better outcomes after immunotherapy in the future (61).

## Author contributions

SJ, W-YS, and YO contributed to the conception and design of the study. All authors contributed to the article and approved the submitted version.
